# Geriatric Syndromes among Patients with Rheumatoid Arthritis: A Comparison between Young and Elderly Patients

**DOI:** 10.4314/ejhs.v32i4.16

**Published:** 2022-07

**Authors:** Fatemeh Niksolat, Zhale Zandieh, Fatemeh Roshani, Samaneh Saghafian Larijani, Hosna Mirfakhraee, Farzaneh Bahadori, Maryam Niksolat

**Affiliations:** 1 Orthopedic Research Center, Faculty of Medicine, Department of Internal Medicine, Mazandaran University of Medical Sciences, Sari, Iran; 2 Iranian Research Center on Ageing, University of Social Welfare and Rehabilitation Sciences, Tehran, Iran; 3 Student Research Committee, Mazandaran University of Medical Sciences, Sari, Iran; 4 Firoozabadi Clinical and Research Development Unit, Iran University of Medical Sciences, Tehran, Iran; 5 PhD candidate Department of Gerontology, University of Social Welfare and Rehabilitation Sciences, Tehran, Iran

**Keywords:** Cognitive Dysfunction, Urinary Incontinence, Aged, Accidental Falls, Arthritis, Rheumatoid

## Abstract

**Background:**

In the general geriatric population, Geriatric syndromes (GSs) predict greater likelihood of hospitalization, increased health care use and cost. The present study aimed to compare GSs among young and elderly patients with rheumatoid arthritis (RA).

**Methods:**

In a cross-sectional study a total of 98 participants, including 65 elderly (≥60 years) and 33 young adult patients (<60 years) with RA who referred to the geriatric and rheumatologic clinic were enrolled. Patients were categorized into three groups (healthy elderly, n=27; elderly with RA, n=38; and young people with RA, n=33). GSs were assessed using mini-mental state exam (MMSE), five-item geriatric depression scale-15 (GDS-15), mini nutritional assessment (MNA), and asking patients about history of falls in the past year. The RA activity in patients was assessed using disease activity for rheumatoid arthritis score-28 (DAS-28) scale, serum ESR (mm/h) level.

**Results:**

There was a statistically significant differences in terms of DAS-28 (2.23±1.01 vs. 0.64±0.97, P=0.025) and ESR (28.10±6.64 vs. 23.09±7.65 mm/h, P=0.042) between healthy elderly and RA elderly patients. Elderly patients with RA were significantly more prone to have cognitive impairment (P=0.002), fall (P=0.005), malnutrition (P<0.001), urinary incontinence (P<0.001), and functional disability (P=0.021) compared to healthy elderlies and young RA patients. The results of binary logistic regression revealed that in elderly RA patients, higher DAS-28 score [odds ratio (OR) = 1.96; 95% CI 1.03, 3.84; P=0.041] was an independent risk factors for the GSs.

**Conclusion:**

The prevalence of some features of GSs were higher in the elderly RA patients than healthy elderly and young RA patients.

## Introduction

Rheumatoid arthritis (RA) is a chronic, progressive autoimmune disease which often leads to impaired functional capacity and poor quality of life (1). It occurs at all ages and in various ethnic groups, but an increase in the prevalence of the disease with increasing age was reported in several studies (1–2). Depending on ethnicity and sex, the average annual incidence rate of elderly-onset RA may vary widely worldwide. According to a study in Spain, the incidence rate of RA per 100,000 elderly people was reported to be 14.5 in females and 9.1 in males (2). In another study carried out in the United States population, the annual incidence rate of RA was approximately 1%, while it was found to be 2% in the elderly population (3). It is known that RA is a high-burden disease and is correlated with a considerable financial burden on patients, their families, and health system (4). A substantial proportion of these costs are due to the increased incidence of clinical conditions named geriatric syndromes (GSs) which are associated with RA disease and are highly prevalent in old age (5). GSs are a range of conditions representing multiple organ impairment including cognitive impairment, depressive symptoms, malnutrition, falls and urinary incontinence (6). To be considered a GS, these conditions must interfere with a person's daily life (7). The etiology underlying the development of GSs is believed to be multifactorial in nature. GSs may result in longer hospitalization stay, higher morbidity and mortality rates in older adults and also an increased health care use and cost (6–7).

With a rapidly aging global population and considering the high prevalence and potential negative consequences of GSs in elderly, early diagnoses and management of GCs in elderly is critical to reduce its burden (7–8). In addition, it has been indicated that RA patients may be at increased risk of GSs (8). These issues demonstrate that investigating the interrelationship between GSs and RA disease among elderly persons is of great importance. To our knowledge, no study has been carried out about GSs in patients with RA in Iran. Therefore, the aim of this study was to compare GSs among healthy elderly and young and elderly patients with RA.

## Methods

This descriptive-analytical study has been conducted in a university hospital affiliated with Mazandaran University of Medical Sciences, Mazandaran, Sari, Iran, from July 2019 to February 2020. A total of 98 persons who referred to the geriatric and rheumatologic clinic of hospital were recruited using purposive sampling. The inclusion criteria for young and elderly RA patients were diagnosis of RA by a rheumatologist, according to the 2010 criteria of the American College of Rheumatology (9). Elderly-onset and young-onset RA has been defined as disease onset at age 60 years or over and beyond 60 years (2). Exclusion criteria were history of stroke or malignancy or hospitalization due to acute illness during 3 month ago. All participants who met the inclusion criteria were categorized into three groups including 27 healthy elderly, 38 elderly RA (≥60 years) and 33 younger RA adults (<60 year) groups.

**Geriatric syndrome assessment**: Assessment of GSs has been made by a gerontologist for all elderly participants. Elderlies who had at least one of the following components were classified in the GSs group. These components were cognitive impairment, depressive symptoms, fall, malnutrition and urinary incontinence (10). Screening of cognitive integrity of all participants was done by mini-mental state exam (MMSE). MMSE is a brief 30-point cognitive screening questionnaire contains 19 items which evaluate the cognitive functions including orientation, concentration and calculation, verbal memory, language, praxis, and visuospatial construction. MMSE scores ≥24, 18–23 and ≤17 was considered as normal cognitive state, mild cognitive impairment and moderate to severe cognitive impairment, respectively (11). A five-item geriatric depression scale-15 (GDS-15) was used to screen depressive symptoms in elderly group. According to this test, scores of 5–8, 9–12 and 13–15 marked the cut-off for the presence of mild, moderate and severe depressive symptoms, respectively (12).

To assess all events, patients were asked whether they have fallen two or more times in the past year. Furthermore, the mini nutritional assessment (MNA) was used to evaluate the risk of malnutrition in participants. Malnutrition was characterized by unintentional body weight loss of 10% in the past half-year (13). In this 30-point questionnaire, scores of 0–17, 18–23 and 24–30 marked the cut-off for the malnutrition, under nutrition and good nutrition, respectively. For evaluation of participants in terms of urinary incontinence, they were asked to determine if they have experienced urinary incontinence in the past year? Have they had urinary incontinence in six separate days?

**Disease activity and functional status assessments**: The Health Assessment Questionnaire Disability Index (HAQ-DI) was used to quantify functional disability and physical dependence in all participants (14). HAQ-DI is a 20-item self-reported functional questionnaire that measures patients' function in eight dimensions rated from 0 to 3. In addition, a rheumatologist was evaluated RA activity in all patients with clinical and laboratory parameters including disease activity for rheumatoid arthritis score-28 (DAS-28), Hb (g/dl) and ESR (mm/h). DAS28 describes severity of rheumatoid arthritis using clinical and laboratory data. It is widely used as a measure of inflammatory disease activity in people with RA during clinical decisionmaking. Previously, it has extensively been validated to monitor disease activity in daily clinical practice (15).

**Sample size calculation**: The estimation of sample size was based on a presumed effect size of 0.3 (6), a statistical power of 95%, and a type I error of 5% using G*Power software, version 3.1.3 with the formula for calculation of sample of correlational studies. The overall proper sample size was found to be 98 participants.

**Statistical analysis**: Data were analyzed using student's t-tests, chi-square test or Fisher's exact test for continuous and categorical variables, respectively. For determining the independent risk factors for GS, binary logistic regressions with backward method were used. Statistical Package for the Social Sciences (SPSS) version 25.0 software (SPSS, IBM Corp., Armonk, NY, USA) was used for data analysis. P-value <0.05 was considered statistically significant.

**Ethical consideration**: The study was performed in accordance with the Declaration of Helsinki and procedures used in this study were approved by the Ethics Committee of Iran University of Medical Sciences, Tehran, Iran (IR.IUMS.FMD.REC.1397.122). In this study, the informed consent was obtained from all participants, after explaining the aim of the study.

## Results

A total of 98 participant including 65 elderly (27 healthy and 38 with RA) and 33 RA young adult patients were included in this study. The mean age of healthy elderlies, adult RA, and elderly RA patients were 64.81±3.89, 46.3±10.25 and 64.46±4.38 years, respectively. Table 1 shows the demographic, clinical characteristics, and GSs components of participants. There are no statistically significant differences for gender, age, Hb (g/dl) and depressive symptoms between young and elderly groups (*P*>0.05). However, statistically significant difference was found for DAS-28 (4.23±1.01 vs. 1.64±0.97, *P*=0.025) and ESR (mm/h) (28.10±6.64 vs. 23.09±7.65, *P*=0.042). In addition, cognitive impairment (27.69 vs. 12.12%, *P*<0.001), fall (27.69 vs. 30.3%, *P*<0.001), malnutrition (70.76 vs. 42.42%, *P*<0.001), urinary incontinence (32.3 vs. 12.12%, *P*<0.001), and HAQ score (2.05±0.61 vs. 0.26±0.11, *P*<0.001) were statistically significant different in both groups (A and B) and elderly group (A) had more possibility of developing this event than the young group (B). As shown in Table 2, compared with elderly healthy subjects (without RA), elderly RA patients significantly were more prone to have cognitive impairment (*P*=0.002), fall (*P*=0.005), malnutrition (*P*<0.001), urinary incontinence (*P*<0.001), and to be physically dependent (*P*=0.021). The results of binary logistic regression revealed that in elderly RA patients, higher DAS-28 score [odds ratio (OR) = 1.96; 95% CI 1.03, 3.84; P=0.041] was an independent risk factors for the GSs.

**Table 1:** Demographic data, laboratory findings, clinical characteristics, and components of GSs in all participants

Variable	Elderly (n=65)	Young (n=33)	P-value
Female	61 (93.84%)	32 (96.96%)	**0.451**
DAS-28	4.23±1.01	1.64±0.97	***0.025***
ESR ,mm/h	28.10±6.64	23.09±76.5	***0.042***
Hb, g/dl	10.06±3.25	13.64±2.89	**0.671**
Components of GSs	47 (72.30)	18 (54.54)	**0.009**
Cognitive impairment	18 (27.69%)	4 (12.12%)	***<0.001***
Depressive symptoms	4 (6.15%)	3 (9.9%)	**0.429**
Fall	18 (27.69%)	10 (30.30%)	***<0.001***
Malnutrition	46 (70.76%)	14 (42.42%)	***<0.001***
Urinary incontinence	21 (32.3%)	4 (12.12%)	***<0.001***
HAQ	2.05±0.61	0.26±0.11	***<0.001***

Data are presented as mean ± S.D. or n (%). The values in the row ‘Components of GSs’ represent the number of participants with any of the geriatric syndromes listed in the rows below

**Table 2:** Comparisons of studied parameters between subjects with and without RA in 65 elderly participants

Variable	Elderly with RA (n=38)	Elderly without RA (n=27)	*P*-value
Age	64.46±4.38	64.81±3.89	**0.817**
Female	36 (94.73%)	25 (92.59%)	**0.555**
Components of GSs	37 (97.36%)	10 (37.03%)	**0.003**
Cognitive impairment	16 (42.10%)	2 (3.70%)	**0.002**
Depressive symptoms	3 (7.89%)	1 (3.70%)	**0.116**
Fall	14 (36.84%)	4 (14.81%)	**0.005**
Malnutrition	35 (92.10%)	9 (33.33%)	**<0.001**
Urinary incontinence	18 (47.36%)	3 (11.11%)	**<0.001**
HAQ	1.56±0.58	0.49±0.03	**0.021**

Data are presented as mean ± S.D. or n (%). The values in the row ‘Components of GSs’ represent the number of participants with any of the geriatric syndromes listed in the rows below

## Discussion

In current study, results have shown that there are no statistically significant differences for gender, age, Hgb (g/dl) and depressive symptoms between elderly and young RA patients. However, statistically significant difference was detected for DAS-28 and ESR (mm/h). In addition, cognitive impairment, fall, malnutrition, urinary incontinence, and disability are statistically significant between elderly patients and young patients so that elderly patients had more possibility of developing these events than the young patients. Besides, our results have shown that higher DAS-28 score was an independent risk factors for the GSs.

Increasing substantial evidence reveals that the persistent systemic inflammation and age-related decline in immune cell functions in RA plays an important role in the development and acceleration of GSs (16). Cognitive functions impairment, as a known GS, includes deficits in attention and concentration, visuospatial and planning functions, mental flexibility, problem solving, and reasoning. Furthermore, adaptive functioning can be influenced by cognitive impairment which considerably hinders conforming to socio-cultural standards and meeting social responsibility. Consistent with current study, in a cross-sectional study performed by Appenzeller et al. on 40 RA patients and 40 healthy controls, it was found that 30% of RA patients were classified with cognitive function impairment compared to 7.5% of healthy controls (17). Additionally, a meta-analysis of 2 cross-sectional studies and 3 cohort studies proved an excess risk of 61% for dementia amongst patients with RA (18–21). The results of a study in Mexico revealed that RA is associated with increased risk of functional impairment in elderly, but not with dementia (22). The pathogenic mechanisms of cognitive impairment in RA remain unclear; however possible causes and risk factors can be multivariable. Although multiple factors are likely to be involved in cognitive impairment of RA patients, RA induced pain is an important factor in this process (23).

It has been previously indicated that in patients with chronic disease, symptoms such as chronic pain are associated with cognitive impairment (24). Despite pharmacological and technological advances, the available evidence suggests that a significant number of RA patients continue to experience pain despite treatment (25). Therefore, it is crucial that clinicians implement appropriate strategies for the optimal management of RA induced pain. Several studies have shown that falls, as major marker of instability, may occurs at any age in patients with RA (26). The current study showed a higher prevalence of falls in elderly RA patients (≥60 years) compared to younger RA patients (<60 years). However, results reported by different studies on the incidence rate of falls in patients with RA versus healthy individuals remain contradictive (27). A study showed almost 27–54% of RA patients with a mean age of 59 years had an increased risk of falling over 1 year (28). In another study, fall incidences in patients with RA has been reported as 68%. In addition, approximately 20% of patients experience multiple falls during a 1 year follow-up (29). The results of another study in Italia showed that about 95% of RA patients are at risk of death due to falls compared to the general population (30). Malnutrition, as another components of GSs in elderly patients can result in increased risk of morbidity and mortality. It has been shown that older patients with RA are more likely to be malnourished (31). This finding is supported by current study and it was found that 92.10% of the elderly patients with RA had malnutrition, while 33.3% of young patients were malnourished. Also, a study by Tański et al. (32) showed malnutrition can adversely affect cognitive function and daily functioning and the severity of frailty syndrome among elderly patients with RA. Further studies are needed to determine the effect of nutritional status improvement on the risk of GSs in elderly RA patients.

Urinary incontinence should not be considered as a normal part of ageing. In particular, it is caused by neurological damage in dementia or stroke, sphincteric damage, and chronic illness and frailty. Arthritis in general has been revealed to be robustly correlated with lower urinary tract symptoms such as incomplete emptying and storage symptoms including increased day time frequency, urgency incontinence, and hesitancy (33). In current study there was a high prevalence of urinary incontinence (47.36%) in elderly patients with RA. Inconsistent with our study, Chen et al. (34) reported no difference regarding the occurrence rate of urinary incontinence amongst elderly compared to younger RA patients. In addition, in a study by Lee et al. (35), lower urinary tract symptoms such as incomplete emptying or voiding dysfunction were equally reported between RA patients and healthy subjects.

In theory, some authors have declared that incontinence in patients with RA may possibly occur as a medication adverse effect (36). Besides, immobility and joint stiffness provide a condition in which patient is unable to reach the toilet or remove their clothing on time (33). Urinary incontinence is a common and distressing complaint in the geriatric population. However, many patients may be embarrassed to seek help or medical treatment because of feelings of shame and fear of others knowing their problems. Therefore, healthcare providers should routinely evaluate elderly persons for urinary incontinence and provide appropriate interventions in prevention, diagnosis and management of this problem.

Current study also showed that there is no statistically difference regarding the depression incidences amongst elderly RA patients compared to younger RA patients and elderly healthy subjects. This result is inconsistent with other studies. In a meta-analysis of 72 studies that included 13189 patients with RA, the frequency of a major depressive disorder was reported to be approximately 17% (37). In another study, depression was reported to affect 25% of RA patients (38). Another study also showed that depression was more frequently encountered in older RA (∼37%) than young individuals (16%) (34). Therefore, in contrast with our study, it seems that prevalence of depression is significantly higher in RA patients. Therefore, using a range of potentially preventive and therapeutic interventions for depression in elderlies is crucial.

Several GS overlap in causes and consequences. For instance, malnutrition and depression may lead to instability, and instability can lead to disability. Dunlop et al. (39) showed that among elderly arthritis patients, disabilities were associated with essential elements of GS such as cognitive dysfunction, depressive symptoms and older age. Results of a study by Dunlop et al. are almost supported by our data which revealed the positive correlation of disability with RA disease and age. Moreover, another study of 100 RA patients has reported that RA disease is positively correlated with functional disability (40), which may leads to GSs. In this study, there are several limitations that should be noted. First, this is a retrospective study and therefore confounding factors which are not considered may create a bias in our results. Therefore, prospective cohort studies are required to elucidate the GSs effect in elderly RA patients. Second, in the present study, elderly people who referred to a teaching hospital were included, which does not necessarily subscribe to the composition of elderlies seen at a community level or primary care setting.

In conclusion, the frequency of some components of GSs was higher in the elderly RA patients compared to the young patients. Therefore, it is important to take action to open new insight into prevention, early detection and appropriate management, of GSs in RA patients. Nevertheless, limited studies in rheumatology seems to consider GSs and thus there is limited information regarding burden of disease among rheumatologists. Future prospective observational follow-up study in larger cohorts will be necessary to increase study power and improve our understanding of the inter-relationship between GS and elderly onset RA.
